# Investigation of gap-graded soils’ seepage internal stability with the concept of void filling ratio

**DOI:** 10.1371/journal.pone.0229559

**Published:** 2020-02-28

**Authors:** Fuhai Zhang, Lei Zhang, Yulong Li

**Affiliations:** 1 Key Laboratory of Ministry of Education for Geomechanics and Embankment Engineering, Geotechnical Research Institute, Hohai University, Nanjing, China; 2 School of Civil and Construction Engineering, Oregon State University, Corvallis, Oregon, United States of America; 3 Tianjin Institute of Geotechnical Investigation Surveying, Nankai District, Tianjin, China; China University of Mining and Technology, CHINA

## Abstract

Gap-graded soils from mountain areas are often used as subgrade filling materials, but problems associated with the gap-graded soils such as large permeability, poor uniformity, and poor seepage stability have to be solved. This article proposes a new terminology “void filling ratio” to study the seepage internal stability of gap-graded soils as subgrade filling materials. Laboratory seepage tests were performed to investigate the effects of compaction degrees of coarse grains, void filling ratios, and clay contents on the internal stability. Laboratory model tests were also performed to verify the findings from the laboratory seepage tests. It was found that the internal stability increased with increase of the void filling ratios, confirmed by both laboratory seepage tests and slope model tests. The increases of both void filling ratio and the clay content were able to change the type of internal instability from piping to the transitional type of internal instability. In laboratory model tests, surface areas lost more fine particles than the deeper area did in the models, but when the void filling ratio was increased, the amount of lost fine particles was significantly reduced. Finally, it was confirmed that void filling ratio was able to effectively describe the internal stability of gap-graded soils subject to different levels of hydraulic gradient.

## 1. Introduction

In highway projects going through mountain areas, gap-graded soils from the same location are often used as subgrade filling materials, because it is really hard to transport large amount of filling material from other locations. However, there are problems using gap-graded soils as subgrade filling materials, such as large permeability, poor uniformity, and poor seepage stability etc. According to [1], gap-graded soils with frame formed by coarse grains are often internally unstable. In this type of soil, fine particles may transport through voids with the water flow, inducing piping failures [2]. Previous studies by other researchers have done many researches focusing on the factors of affecting internally stability, such as particle size distribution[e.g. 3–7], hydraulic conditions [e.g. 8,9], and confining pressure and seepage forces [e.g. 10]. With laboratory tests, other researchers investigated the internal instability from aspects such as seepage under different loading scenarios [e.g. 11–13], the effects of hydraulic loading history [14], and spatial scale effects on internal instability [15]. Additionally, numerical simulations [e.g. 16,17] and micro-scale images [e.g. 18,19] were also used to study the internal instability. In order to better investigate the internal instability of gap-graded soils due to seepage forces with fine particles moving through void spaces formed by large particles, a new terminology “Void Filling Ratio” is proposed as:

$$VFR=\frac{V_{f}}{V_{v}}\times 100$$ (1)

where: *VFR* is the Void Filling Ratio; *V*_*f*_ is the volume of the fine particles; *V*_*v*_ is the volume of the void. Performing hydraulic tests were always an excellent approach to determine whether a granular material is potentially unstable or not [3]. In this work, series of laboratory seepage tests were performed on soils with different compaction degrees of coarse grains, different void filling ratios, and different amount of clay particles. In addition, a laboratory model of road subgrade filled with gap-graded soils was built with different void filling ratios to study the amounts of fine particles flowing out.

## 2. Laboratory seepage test program

### 2.1 Seepage test device

**Fig 1:**
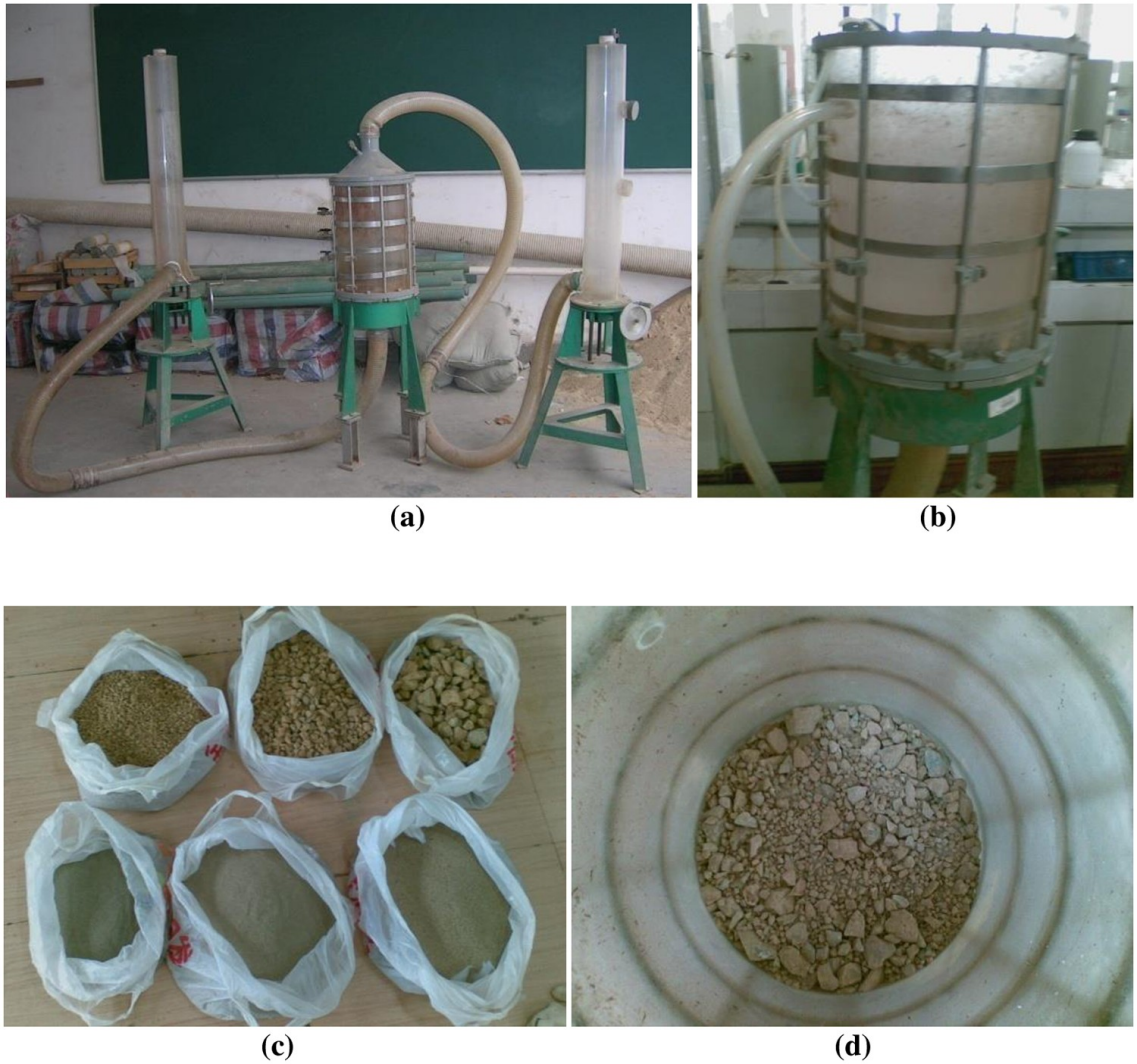
The test device and sample materials in seepage test: (a) device overview; (b) sample container; (c) sample materials with different particle sizes; (d) material filling process.

In the seepage test, a set of device was built in the geotechnical laboratory in Hohai University and shown in Fig 1(a). With this set of device, the hydraulic gradient could be accurately controlled, the amount of seepage fluid can be accurately measured, and the movement of soil particles in the water flow could also be directly observed. The three main parts of the device were the water supplier, the sample container shown in Fig 1(b), and the seepage fluid collector with volume measurement. The internal diameter of the sample container was 30 cm, and the sample may be prepared with the height of 0–40 cm. Water flowed from the bottom to the top of the container. A filter was added to the bottom to prevent the loss of fine particles form the bottom. The top surface of the sample was free. The hydraulic pressure in the sample container was measured every 10 cm from the bottom to the top.

### 2.2. Seepage testing materials and sample preparation

Fig 1(c) shows the testing materials which was collected from the section of K109-K119 on Road Nanyou in Guangxi in China. The material tested in this sample was a gap-graded mixture of coarse grains and fine particles which were prepared separately. The particle diameters in the coarse grains were from 5 mm to 40 mm, and the fine particles had diameters smaller than 1 mm. Fig 1(d) shows the sample in the container.

In the very first, it can be assumed that there are only coarse grains in the soil, and the void ratio (*e*_*c*_) is calculated as:

$$e_{c}=\frac{G_{s}\times \rho_{w}}{\rho_{d}}-1$$ (2)

where: *G*_*s*_ is the specific gravity of the soil particles; *ρ*_*w*_ is the density of water; *ρ*_*d*_ is the dry density. The volume of void space between coarse grains (*V*_*vc*_) can be calculated as:

$$V_{vc}=V_{sc}\times e_{c}$$ (3)

where: *V*_*sc*_ is the volume of the solid of the coarse grains. Therefore, the mass of the fine particles needed (*m*_*c*_) is:

$$m_{c}=V_{vc}\times VFR\times \rho_{f}$$ (4)

where: *ρ*_*f*_ is the density of the fine materials in the voids when the void ratio is *e*_0_ calculated with the relative density of 1/3 which is the boundary between the loose condition and the medium dense condition.

**Fig 2:**
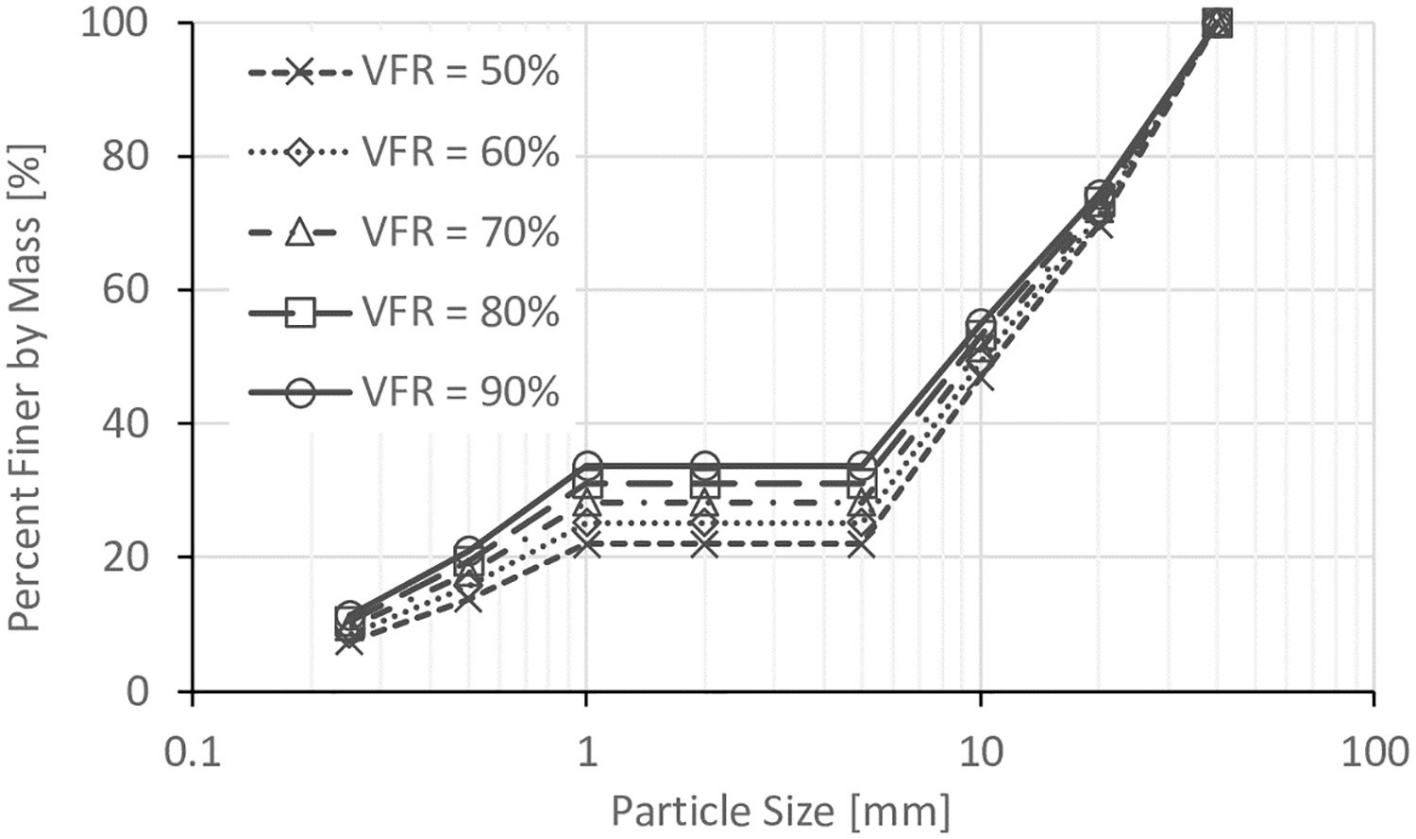
Particle size distribution for the sample with *D*_*c*_ = 80%.

**Fig 3:**
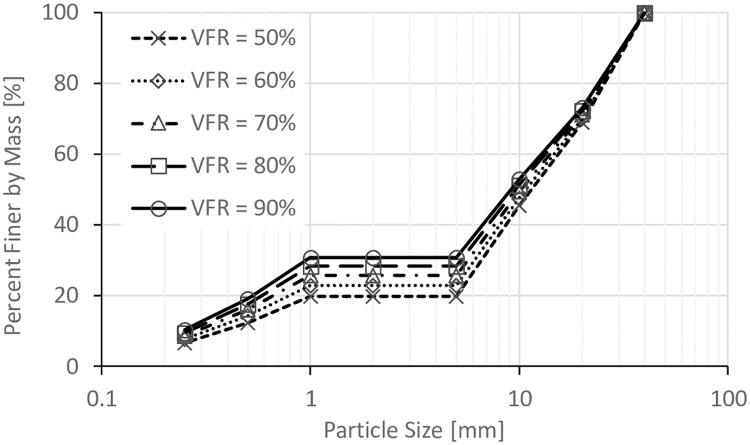
Particle size distributions for samples with *D*_*c*_ = 85%.

**Fig 4:**
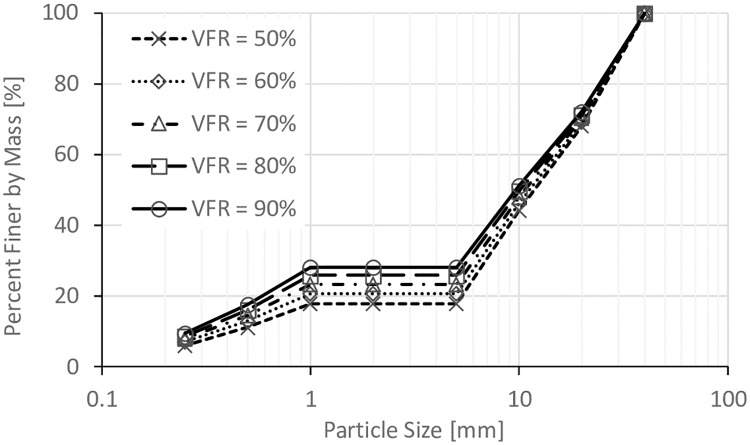
Particle size distributions for samples with *D*_*c*_ = 90%.

**Fig 5:**
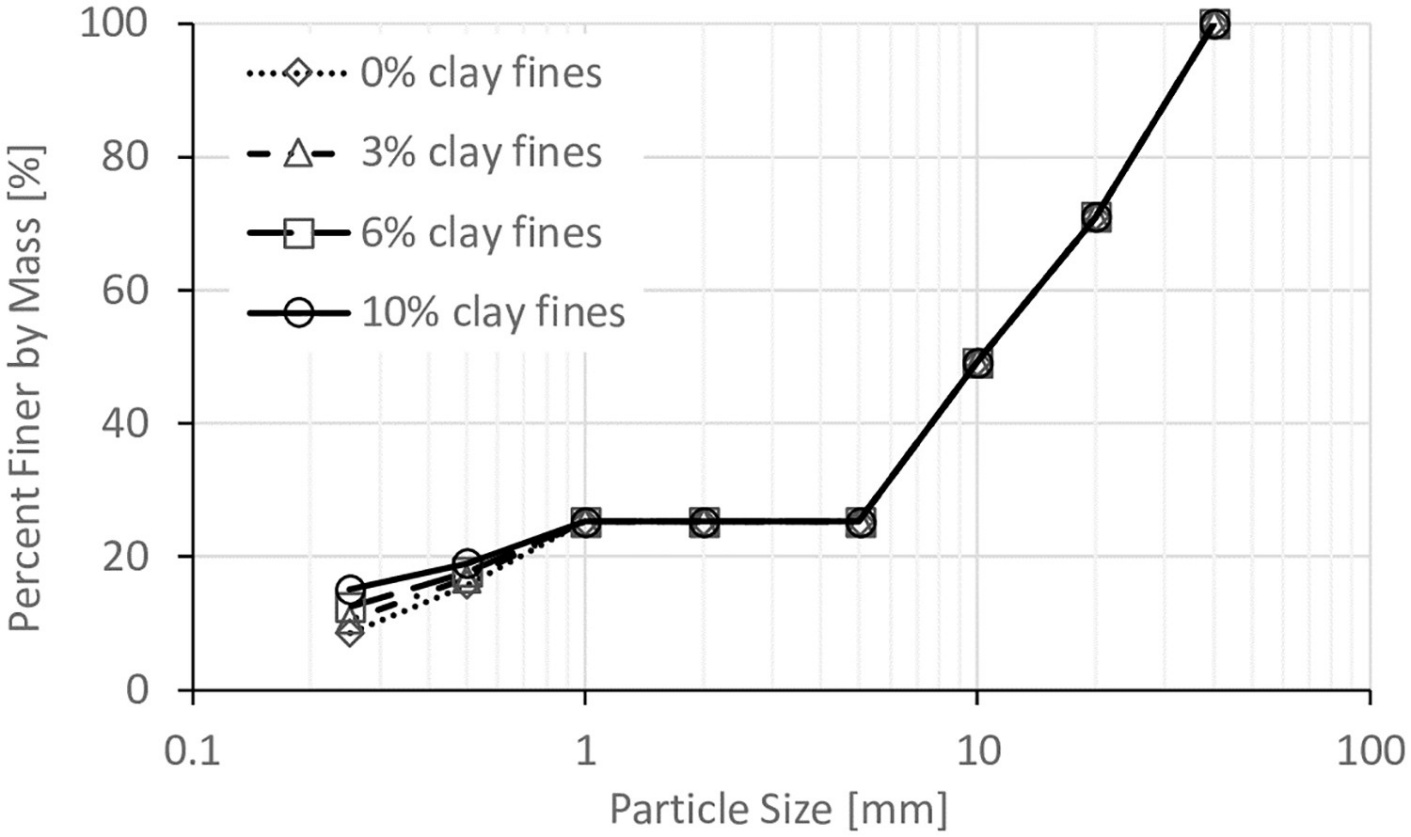
Particle size distributions for samples with different amount of clay fines.

The size of void space is directly related to the degree of compaction of the coarse grain formed soil frame (*D*_*c*_) (where: *D*_*c*_ = dry density of coarse grains/ maximum dry density of coarse grains), so three *D*_*c*_ values (80%, 85%, and 90%) were used in this work. Five values of *VFR* (50%, 60%, 70%, 80%, and 90%) were used to study the effect of *VFR*. In addition, in order to study the effect of clay fines proportion, soils with 0%, 3%, 6%, and 10% of clay fines were prepared with *VFR* = 60% and *D*_*c*_ = 80%. The particle size distributions for all these samples are shown in Figs 2, 3, 4 and 5.

## 3. Laboratory seepage test results and discussion

### 3.1. Effects of *D*_*c*_

**Fig 6:**
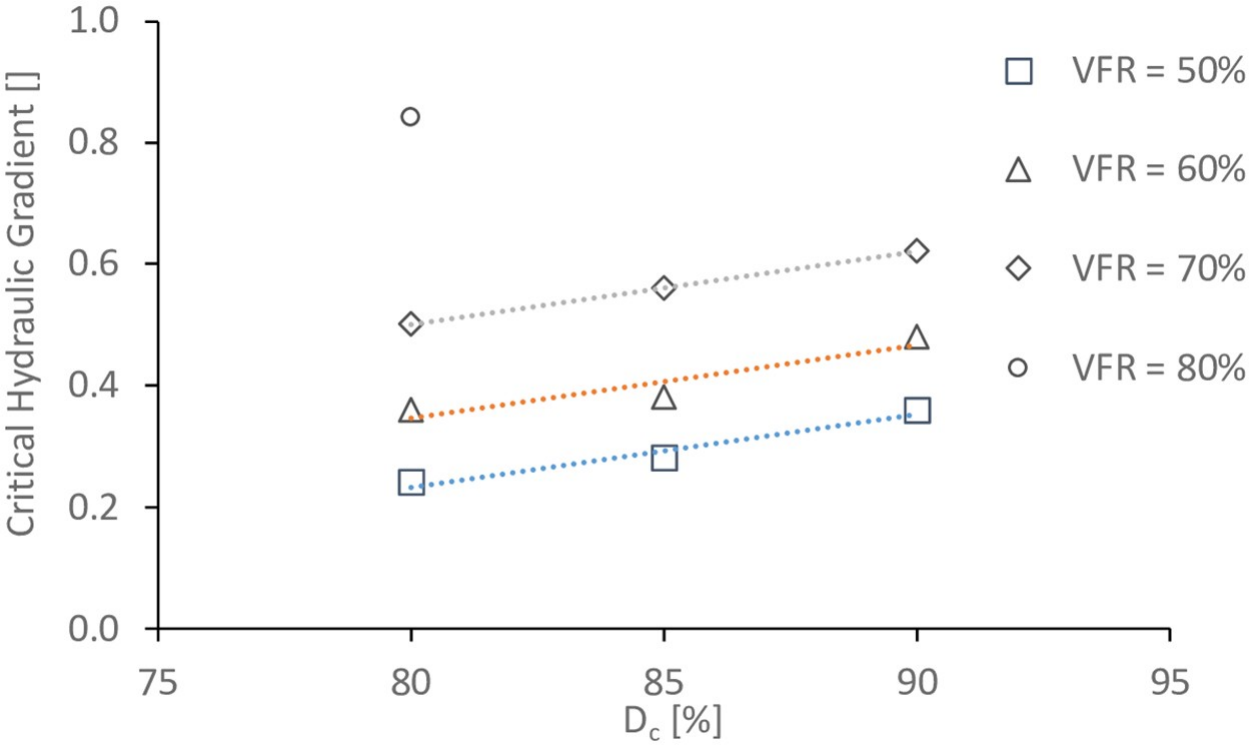
The effect of *D*_*c*_ on the critical hydraulic gradient for specimens with different VFR.

Soils with small values of *D*_*c*_ have large void spaces and weak inter-locking between particles, so fine particles in the voids may be easily flowed away and the shear strengths will be small. Therefore, it is critical to study the effect of *D*_*c*_ on the internal stability for gap-graded soils. The start of internal instability is directly related to the critical hydraulic gradient which could be defined as the hydraulic gradient at which fine particles start to flow out, and this may be directly observed as “soil boils” [20] or other types of soil flowing out [9]. Referring to the analyzing methods from literatures [e.g. 21], the critical hydraulic gradients at different values of *D*_*c*_ for specimens with different VFR values are plotted in Fig 6. Trending lines are added to the data sets with VFR = 50%, 60%, and 70%. It can be seen that these three lines are parallel to each other. With the increase of *D*_*c*_ values, the critical hydraulic gradient is increasing dramatically with the same VFR value. For the tests with VFR = 80%, when the values of *D*_*c*_ are larger than 80%, the critical hydraulic gradients which are not plotted in Fig 6 are even higher than 1.5.

### 3.2. Effects of VFR

**Fig 7:**
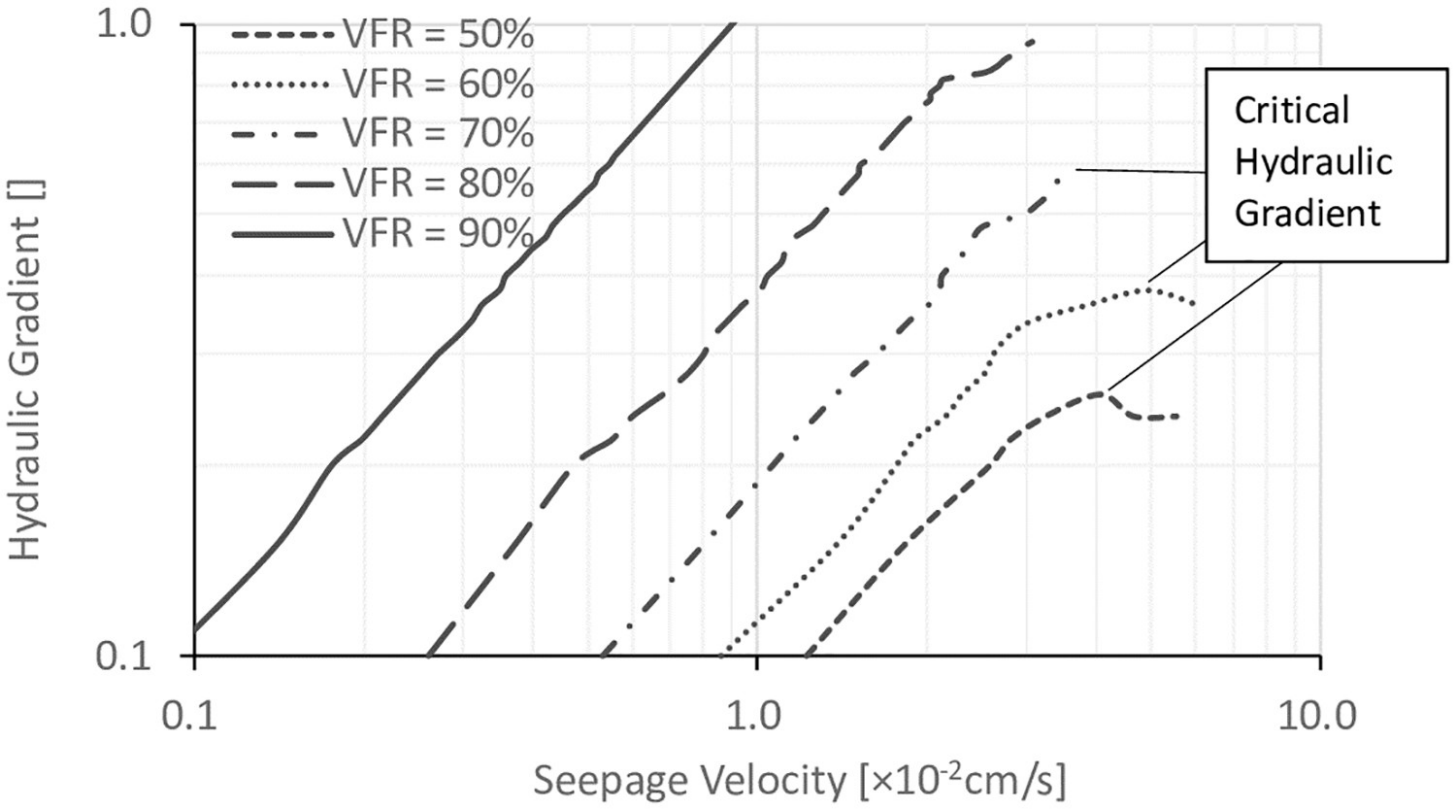
The relationship between the hydraulic gradient and the seepage velocity for specimens with different VFR values and *D*_*c*_ = 80%.

**Fig 8:**
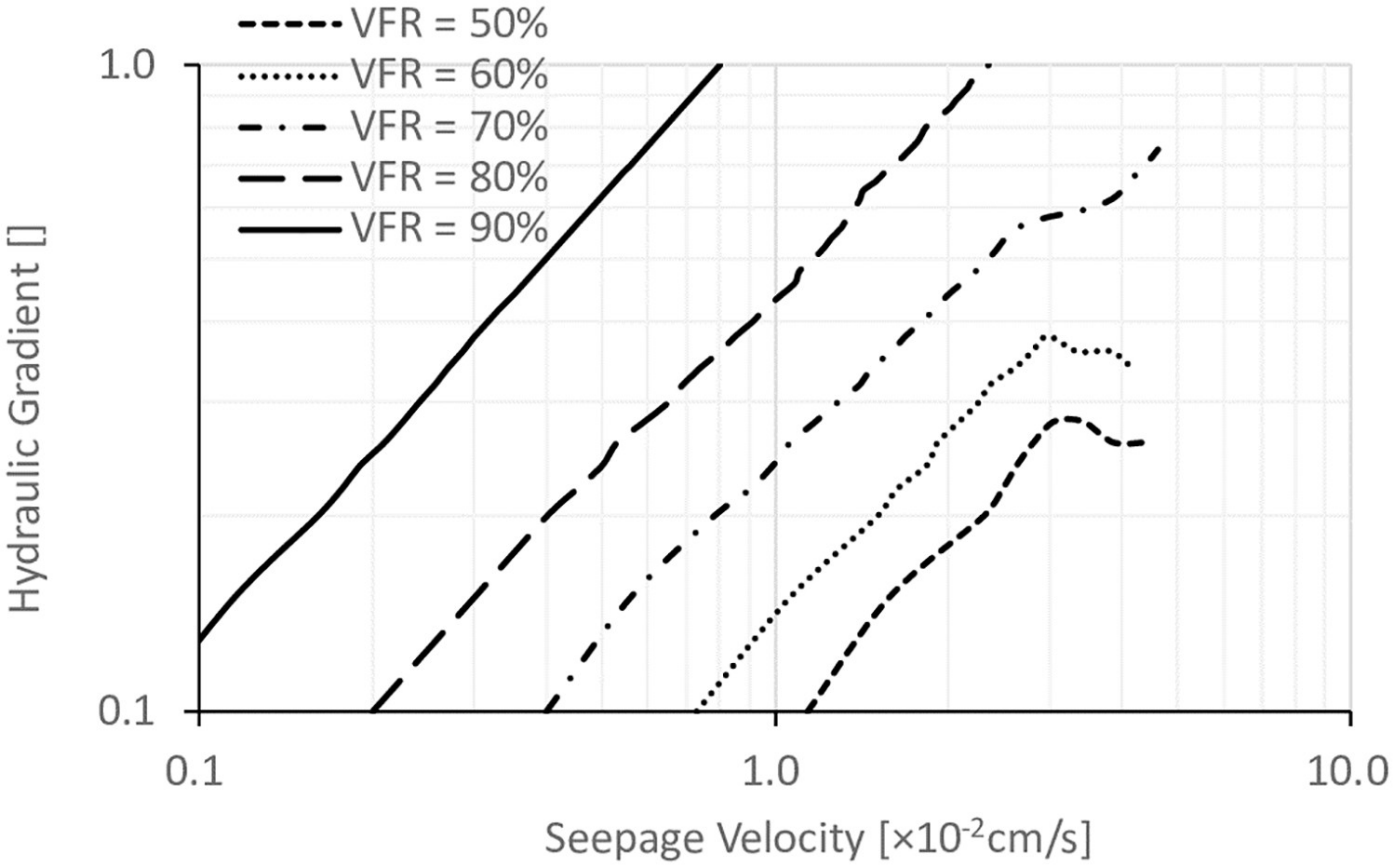
The relationship between the hydraulic gradient and the seepage velocity for specimens with different VFR values and *D*_*c*_ = 85%.

**Fig 9:**
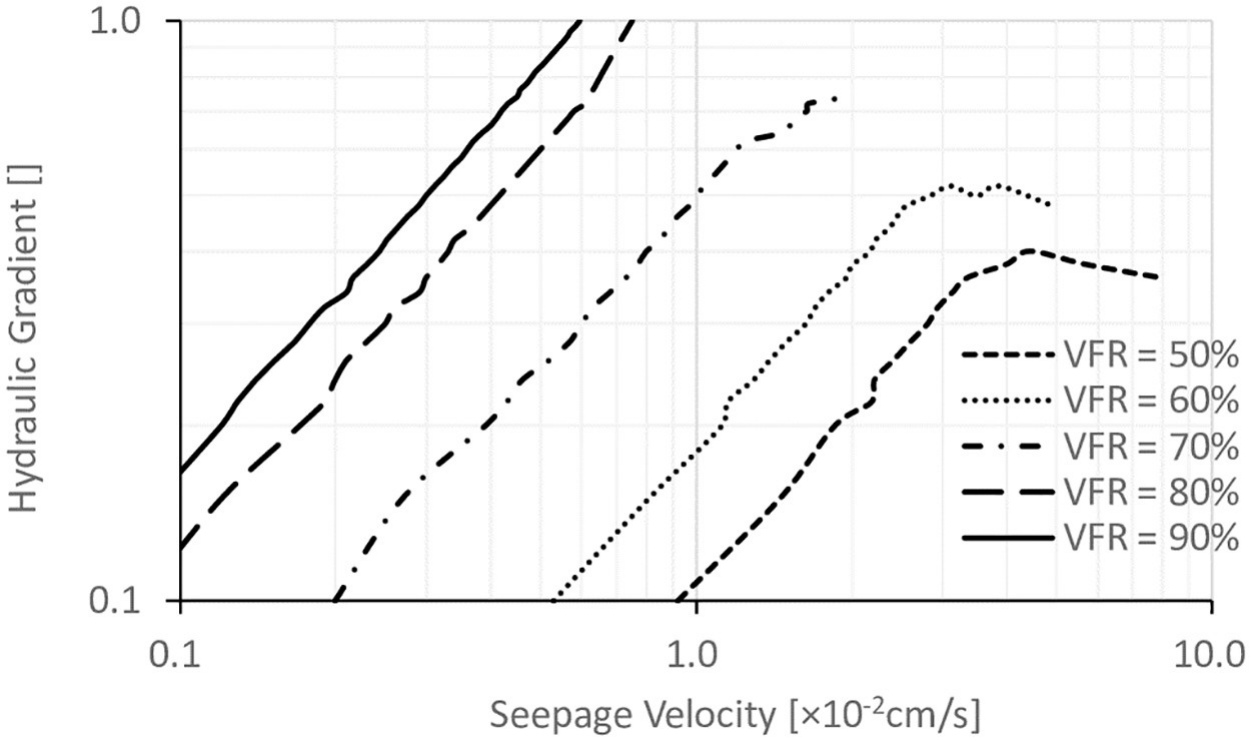
The relationship between the hydraulic gradient and the seepage velocity for specimens with different VFR values and *D*_*c*_ = 90%.

In order to study the effect of VFR on the internal stability, hydraulic gradients are plotted versus seepage velocity defined as the ratio of volume of flowing-out water to time interval between readings, which is shown in Figs 7, 8 and 9 with different *D*_*c*_ values. The critical hydraulic gradients were defined at the state when “sand boils” started to occur, which could be observed during testing. It can be seen that at a certain *D*_*c*_, with the increase of the VFR values, the hydraulic gradients were significantly increased. When VFR values were higher than 70%, there was no failure happening. When VFR values were smaller than 70%, with the increase of seepage velocity, the hydraulic gradients were increased first, reached a peak later, and finally were decreasing after the peak, corresponding to the process in which fine particles were gradually flowed out and consequently, soil frame can not resist higher hydraulic gradients. [22] classified sandy soils based on the types of seepage instability-piping, transition, and soil-flow. It was stated that when piping occurred, fine particles were flowed out, and hydraulic gradients started to decrease finally, which was corresponding to the bending down in Figs 7, 8 and 9 when VFR values were smaller than 70%. In the transition type of seepage instability, piping occurred first, and then, fine particles stopped flowing out with the increase of hydraulic gradient until failure occurred on the soil surface, corresponding to the failure type when VFR equaled to 70% in Figs 7, 8 and 9. In the soil-flow type of seepage instability, soil particles uplifted at the same time which was common with silty soils and not found in this work. Therefore, the soil samples in this work can be classified with this method and shown in Table 1. In order to study the piping type further, Table 1 also lists the fines content (P_f_), dry density (ρ_d_), critical hydraulic gradient (i_cr_), and the coefficient of permeability (k). By summarizing the data in Table 1, it can be concluded that when P_f_ was smaller than 25%, piping occurred. When P_f_ was in the range of 25%–30%, the transitional seepage instability was the major type of instability. However, there was also exception. For example, when *D*_*c*_ was equal to 90%, and VFR equal to 70%, it was transitional seepage instability, but the value of P_f_ was smaller than 25%. In addition, it can be found that the boundary between piping and transitional seepage instability was around 1.9 g/cm^3^ in terms of ρ_d_, and about 0.5 in terms of i_cr_.

**Table 1:** Classification of soil samples.

*D*_*c*_ (%)	VFR (%)	50	60	70	80	90
80	P_f_ (%)	21.96	25.24	28.26	31.05	33.62
	ρ_d_ (g/cm^3^)	1.74	1.82	1.9	1.97	2.05
	i_cr_	0.24	0.36	0.5	0.84	N/A
	k (cm/s)	1.22×10^−1^	8.6×10^−2^	5.3×10^−2^	2.6×10^−2^	1.0×10^−2^
	Instability Type	Piping	Piping	Transition	Transition	Stable
85	P_f_ (%)	19.85	22.91	25.75	28.38	30.83
	ρ_d_ (g/cm^3^)	1.8	1.87	1.95	2.02	2.09
	i_cr_	0.28	0.38	0.56	N/A	N/A
	k (cm/s)	1.14×10^−1^	7.3×10^−2^	4.0×10^−2^	2.0×10^−2^	8.0×10^−3^
	Instability Type	Piping	Piping	Transition	Stable	Stable
90	P_f_ (%)	17.88	20.71	23.36	25.83	28.15
	ρ_d_ (g/cm^3^)	1.86	1.93	2.00	2.06	2.13
	i_cr_	0.36	0.48	0.62	N/A	N/A
	k (cm/s)	9.2×10^−2^	5.3×10^−2^	2.0×10^−2^	8.0×10^−2^	6.0×10^−2^
	Instability Type	Piping	Piping	Transition	Stable	Stable

### 3.3. Effects of clay content

**Fig 10:**
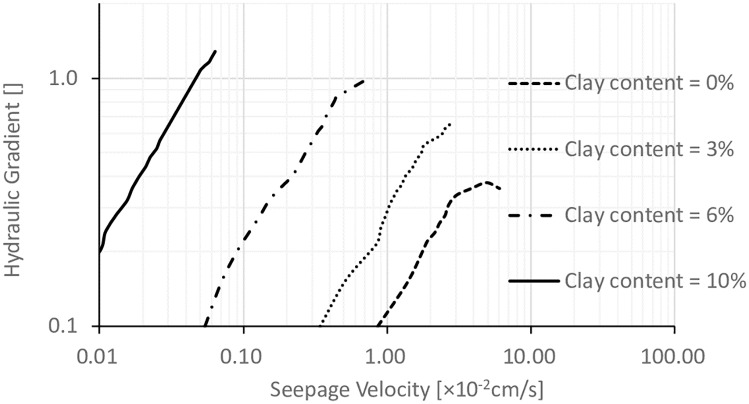
The relationship between the hydraulic gradient and the seepage velocity for specimens with different clay contents.

[23] stated that most of the clay materials in real projects were non-dispersive clay which had high cohesion, and the soil-flow type of seepage instability was the major type of seepage instability. With the installation of filter, the critical hydraulic gradient could be significantly increased, and around 4 ~ 5 for typical clay soils. In addition, [24] stated that the permeability of coarse-grained soils was governed by both the highly permeable coarse grain portion and the fine grain portion with low permeability. The seepage instability of coarse-grained soils was due to the loss of fine particles in the voids between large particles, with the increase of void space volume and coefficient of permeability. Clay materials typically has very high cohesion with the coefficient of permeability smaller than 10^-5^cm/s which is so much smaller than the coefficient of permeability of the materials used in this work. Therefore, it is critical to study the effect of clay contents on the permeability. Fig 10 shows the hydraulic gradients for specimens with *VFR* = 60%, *D*_*c*_ = 80%, and different contents of clay particles. It can be seen that when the clay content was zero, the seepage instability was in the form of piping, and when the clay content was equal to or higher than 3%s, the seepage instability was change to the form of transitional seepage instability. The values of other parameters in the tests are listed in Table 2. It can be seen that with the increase of clay contents, the values of i_cr_ were increased dramatically, and the values of the coefficient of permeability were reduced on the level of exponent which was associated with the change of seepage instability type.

**Table 2:** Results of the seepage tests with different clay contents.

Parameters	Clay content (%)			
	0.0	3.0	6.0	10.0
P_f_ (%)	25.25			
ρ_d_ (g/cm^3^)	1.82			
i_cr_	0.36	0.55	0.84	1.12
k (cm/s)	8.6×10^−2^	3.4×10^−2^	5.4×10^−3^	4.7×10^−4^
Instability Type	Piping	Transition	Transition	Transition

## 4. Laboratory model test

In the piping type soil samples, fine particles will be flowed out with seepage water flow, reducing the seepage stability and subsequently causing problems such as pavement settlement and slope failure. Therefore, it is important to study the way of fine particles flowing out with seepage forces.

### 4.1. Model properties

**Fig 11:**
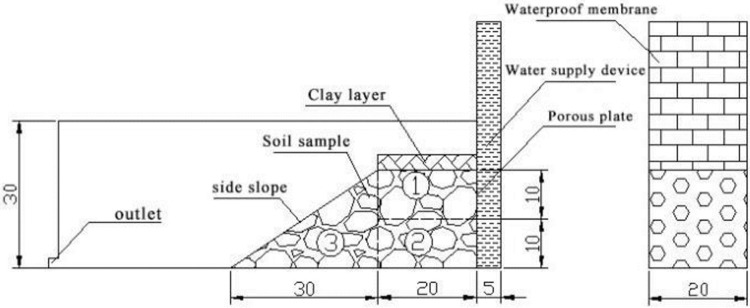
Schematic diagram of the laboratory model. The unit of length scales is centimeter, and the model was divided into three areas numbered with circled numbers.

Four laboratory slope models were built and filled with gap-graded soil materials, shown in Fig 11 with dimensions of the model. The materials used in this model had the same *D*_*c*_ = 85% with different VFR values (50%, 60%, 70%, and 80%). The model was built as a soil slope with a layer of clay on the top to prevent seepage from the top. A water supply wall was set on the back of the slope to provide the same hydraulic gradient field on the direction of the length of the slope perpendicular to the paper. Water and fine particles flowed out from the slope were firstly stored in the water container in front of the slope with a vent on the left side, so fine particles could be settled down in the container and weighted later after the test.

In the test, water level in the water supplier was first kept 5 cm above the model, so the hydraulic gradient was 0.1. 24 hours later, the fluid was collected for the past 10 min and the volume was measured. The same type of measurement was performed every 6 hours for totally three times. Fine particles were collected, dried and weighted. If the seepage flow was relatively stable and there was no failure, the water level in the water supplier would be increased by 1 cm. Then the fluid was collected and tested again after 24 hours. The same procedure was repeated until failure occurred or the slope was continuously stable.

### 4.2. Test results and analysis

**Fig 12:**
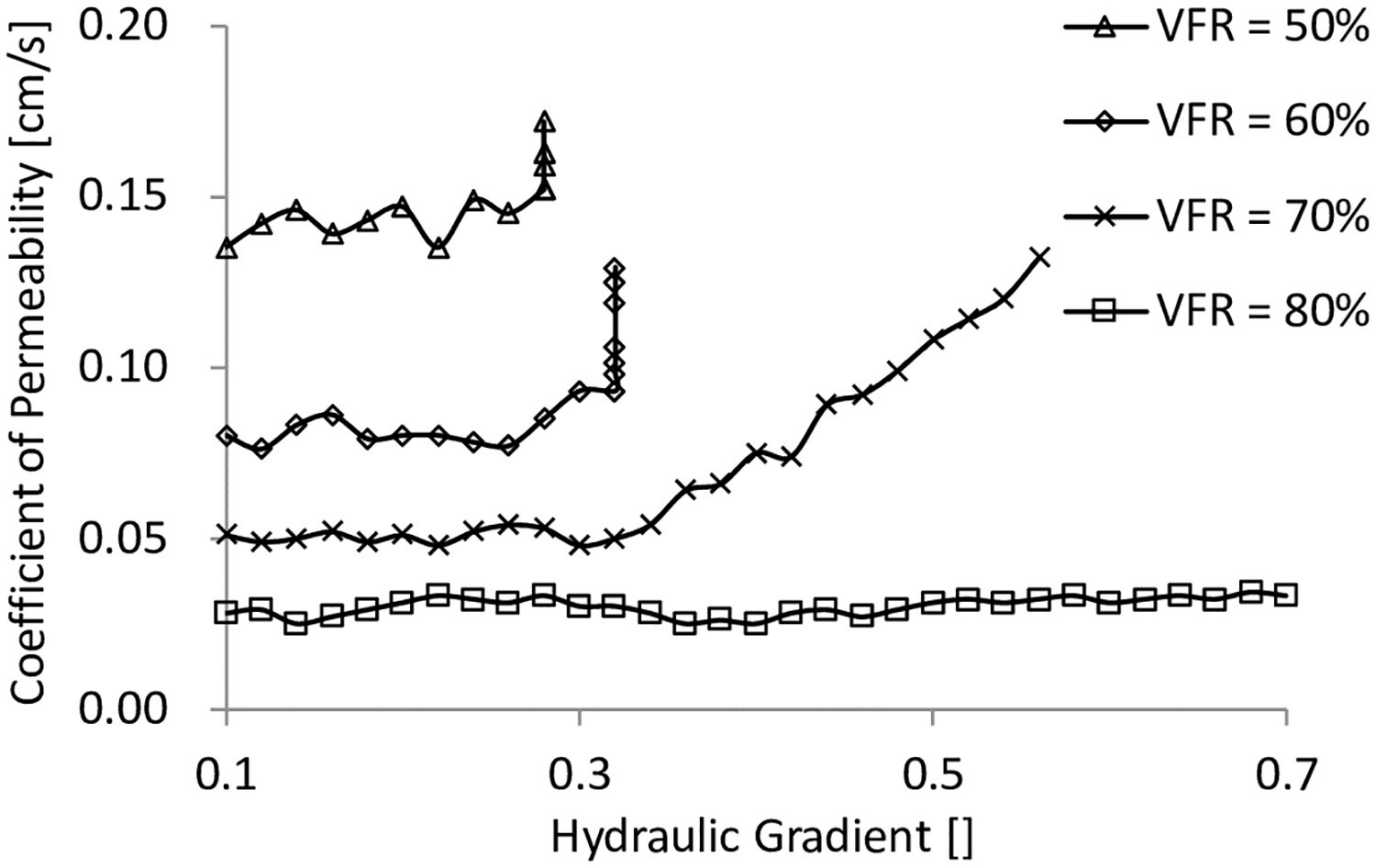
The relationship between the coefficient of permeability and the hydraulic gradient.

**Fig 13:**
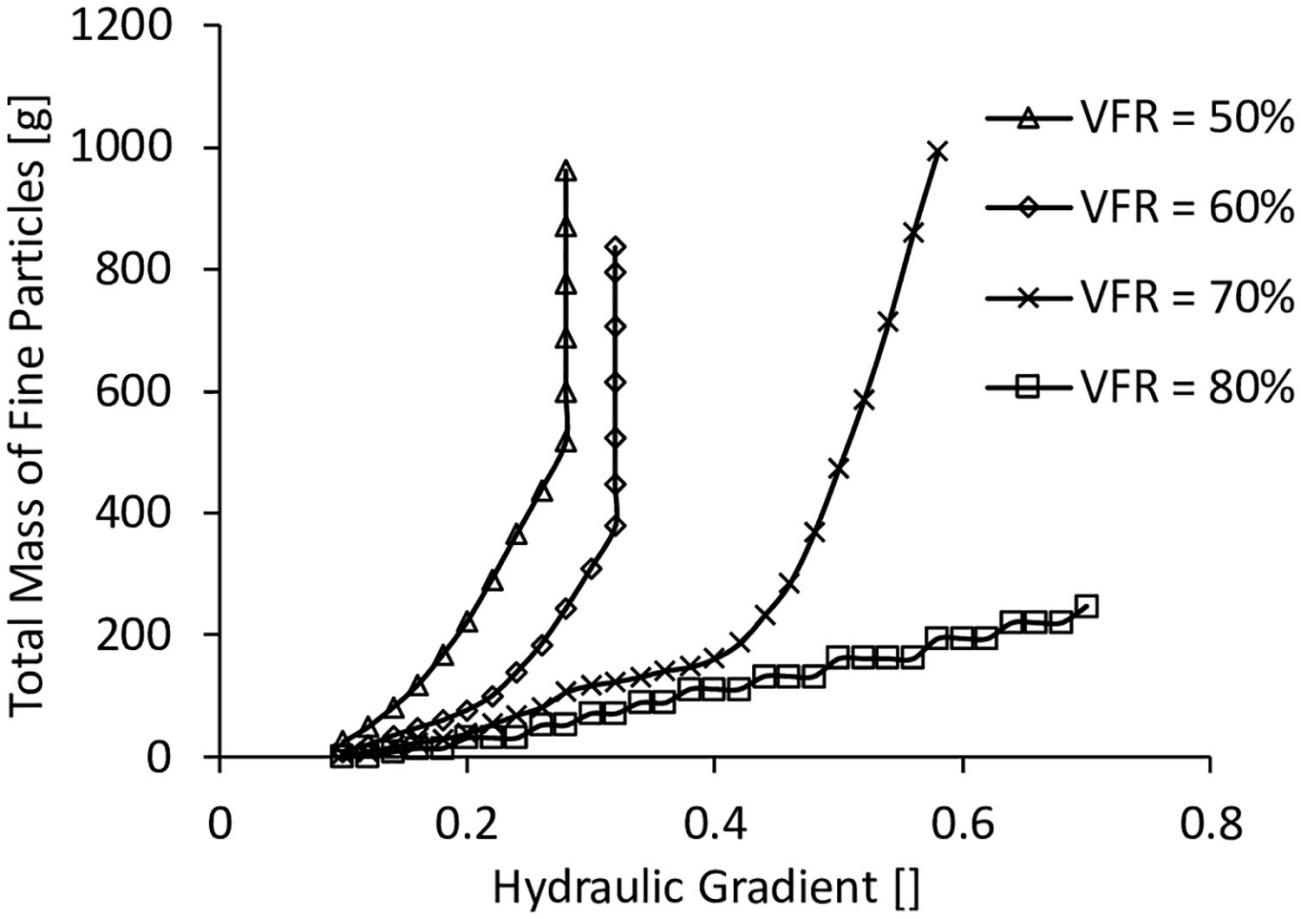
The accumulated total mass of fine particles in the water collector.

The coefficients of permeability with different hydraulic gradients are plotted in Fig 12. The accumulated total mass of fine particles in the water collector is shown in Fig 13. It can be seen that when the VFR values were equal to 50% and hydraulic gradients were smaller than 0.28, the coefficients of permeability were all around 0.14 cm/s, but there was already fine particles flowing out and accumulated. When hydraulic gradients reached 0.28, the coefficients of permeability and the accumulated total mass of fine particles were increasing significantly even without any change of hydraulic gradient, which was a sign of internal instability. When VFR values were equal to 60%, similar dramatic increases of the coefficient of permeability and the accumulated total mass of fine particles occurred at the hydraulic gradient = 0.32. At VFR = 70%, the coefficient of permeability was almost constant, and the accumulated total mass of fine particles were increasing slowly when the hydraulic gradients were smaller than 0.33. When the hydraulic gradients were larger than 0.33, the coefficient of permeability started to increase, and the accumulated total mass of fine particles increased much faster but not like the sharp increase when VFR was smaller than 70%, indicating that the internal structure was more stable than those with VFR values smaller than 70%. When VFR values were equal to 80%, the internal structure was even much more stable with the coefficient of permeability constant, and the accumulated total mass of fine particles increased very slowly. Finally, it can be concluded that the increase of VFR from 50% to 80% significantly increased the internal stability of the material used in this work.

**Fig 14:**
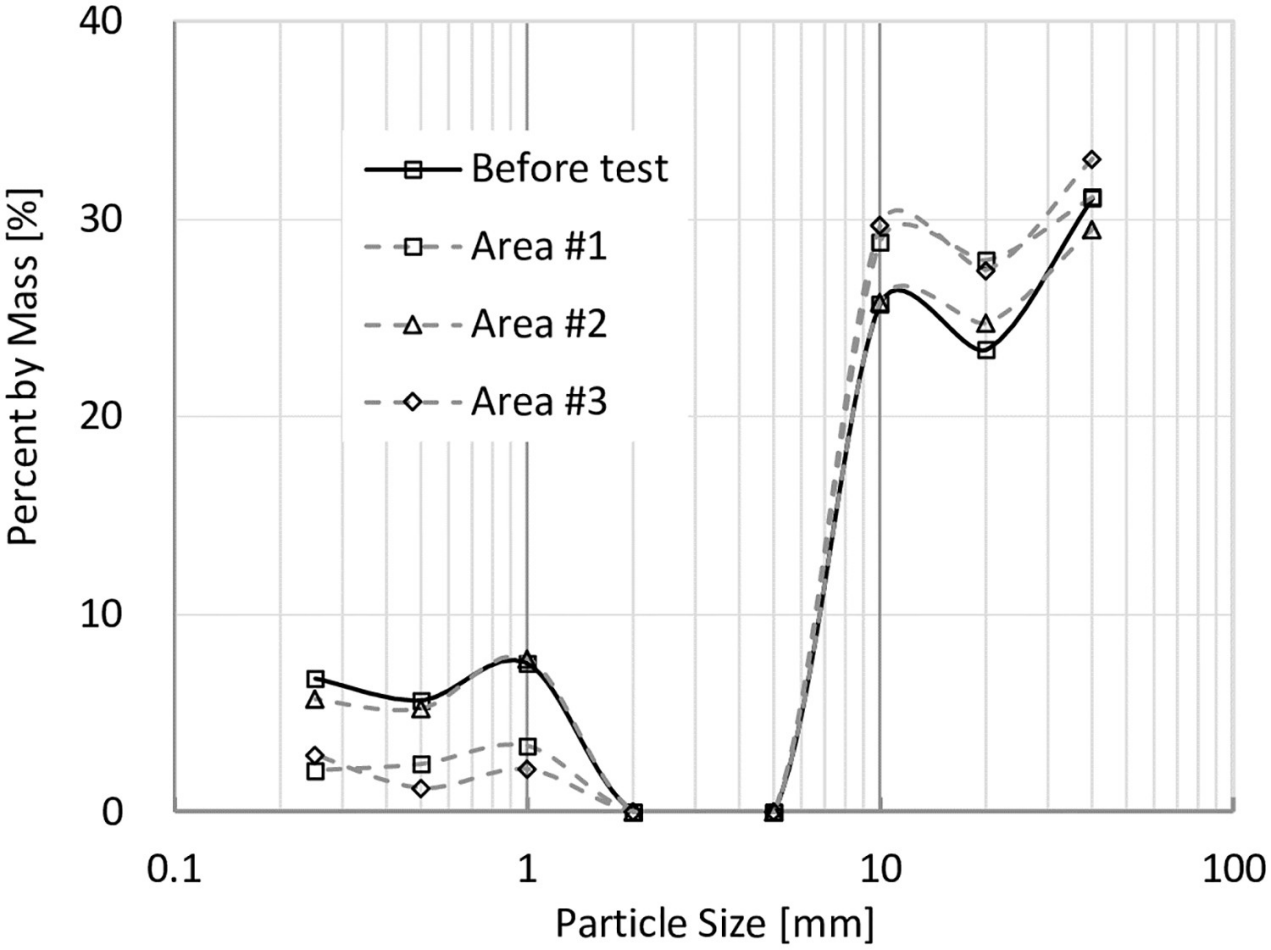
Frequency of particles with different sizes in each area of the model with VFR = 50%.

**Fig 15:**
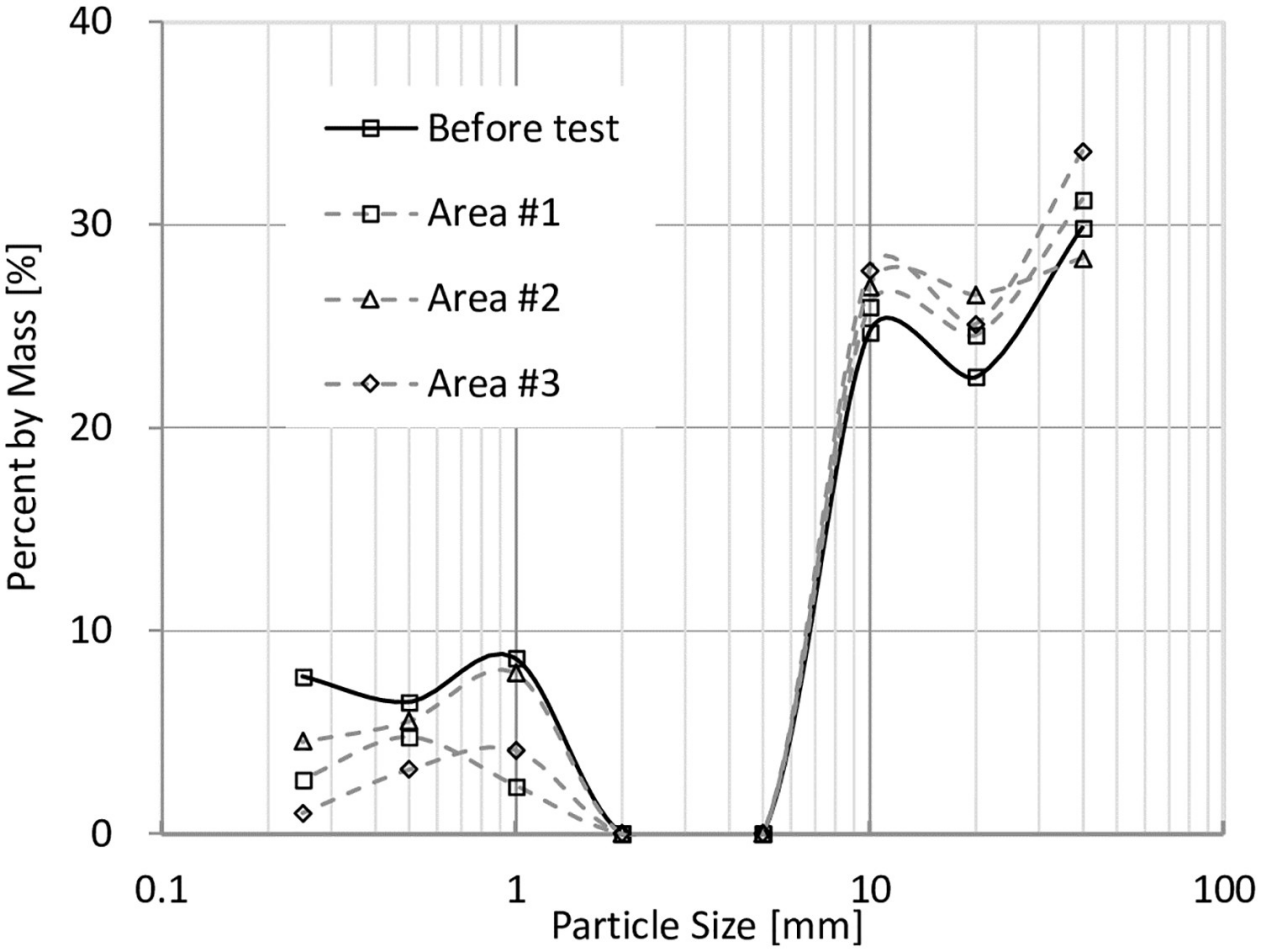
Frequency of particles with different sizes in each area of the model with VFR = 60%.

**Fig 16:**
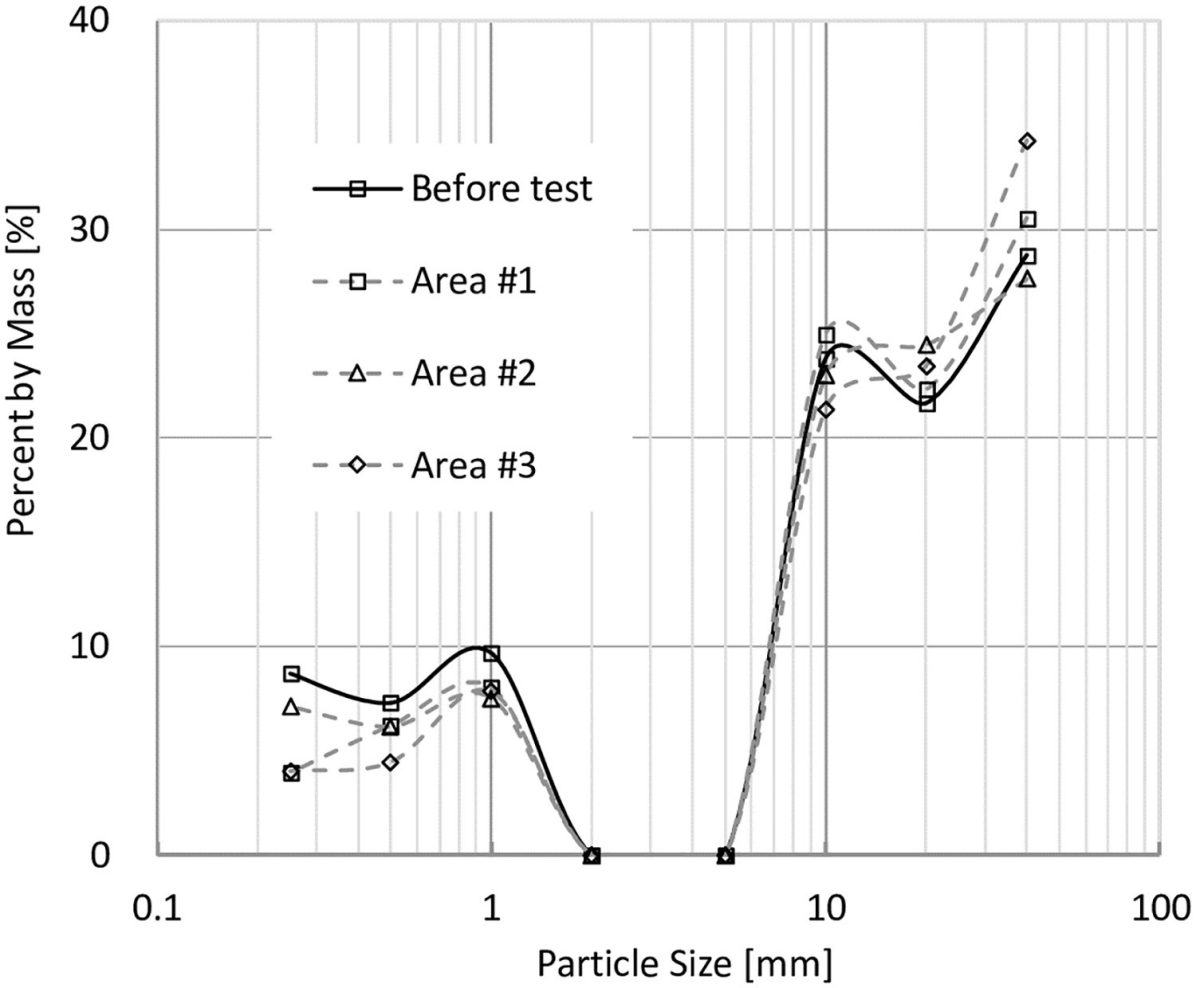
Frequency of particles with different sizes in each area of the model with VFR = 70%.

**Fig 17:**
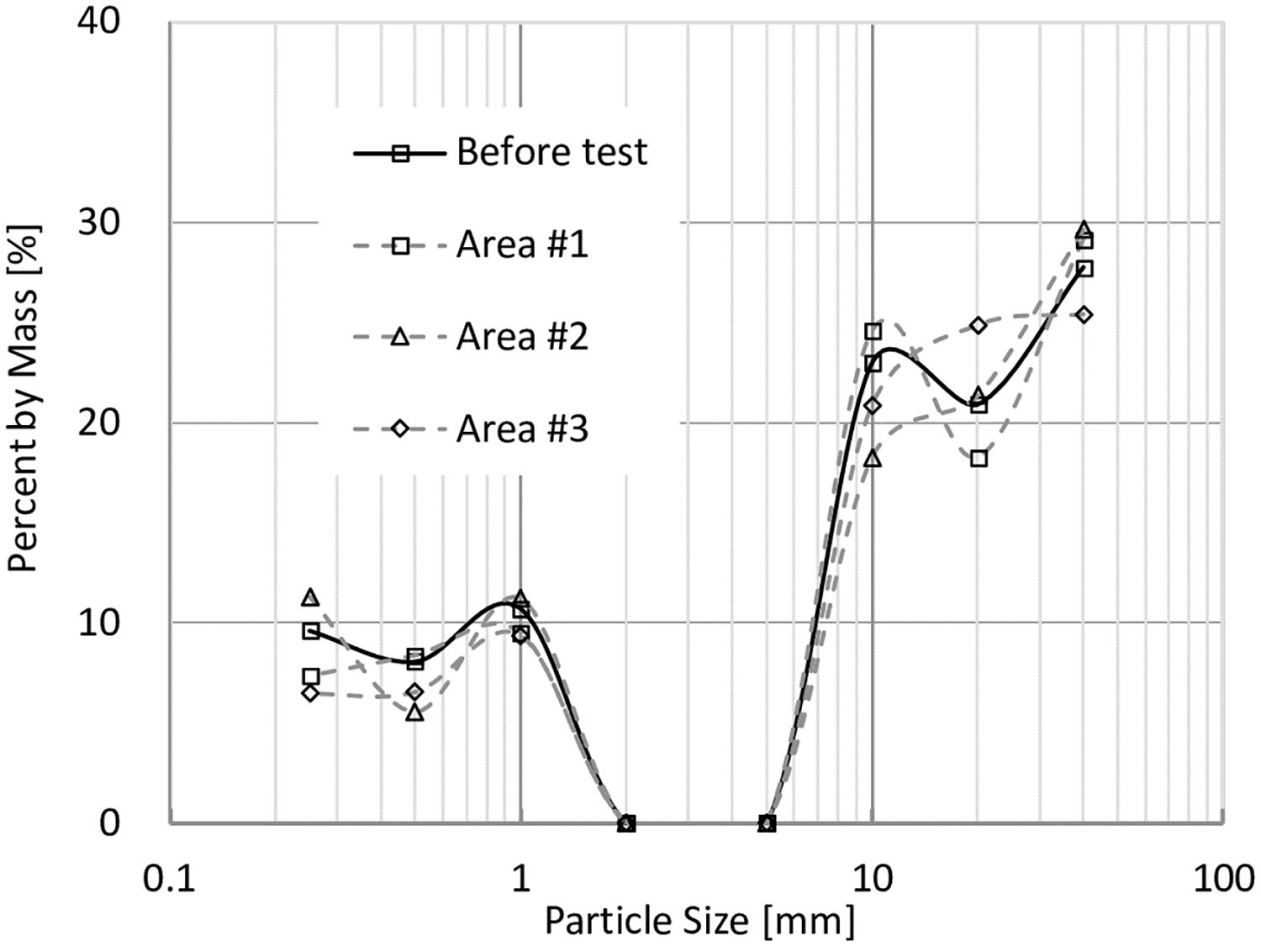
Frequency of particles with different sizes in each area of the model with VFR = 80%.

In order to study the fine particle distributions in the models before and after tests, models were divided into three parts shown in Fig 11, and the particle size distributions are shown in Figs 14, 15, 16 and 17. In Figs 14 and 15, it can be seen that when VFR = 50% and 60%, Area #1 and Area #3 lost much more fine particles than Area #2 did. In Figs 16 and 17, when VFR = 70% and 80%, it can be seen that Area #1 and Area #3 still lost more fine particles than Area #2 did, but the difference of the amount of fine particle loss between areas were not as huge as that when VFR = 50% and 60%. Therefore, it can be concluded that the increase of VFR values increased the internal stability in these models.

## 5. Conclusions and recommendations

In this paper, a new terminology “void filling ratio” was created to effectively study the internal stability of gap-graded soils subjected to different hydraulic gradient. Laboratory seepage tests, and model tests were performed. Important conclusions and insights are as follows:

1. The increase of VFR values increased the internal stability, which was confirmed by both laboratory seepage tests and slope model tests. *D*_*c*_ is also able to increase the internal stability of gap-graded soils.
2. The increase of VFR could change the type of internal instability from piping to the transitional type of internal instability.
3. The increase of clay content in the fine material also increased the internal stability.
4. In laboratory model tests, when the hydraulic gradient was relatively small, the increase of hydraulic gradient did not change the permeability though the accumulated total amount of fine particles was increasing.
5. In laboratory model tests, surface areas lost more fine particles than the deeper area did in the models. However, when the VFR was increased, the amount of lost fine particles was significantly reduced.

Though important findings were observed based on the concept of VFR, transportations of fine particles were not directly viewed and quantified. New tools like the discrete element numerical simulation may be used later to study the particle transportation and internal instability in a more detailed way. Long-term internal stability was not studied in this work, and may be investigated in the future work.
