# Correction: Investigation of gap-graded soils’ seepage internal stability with the concept of void filling ratio

**DOI:** 10.1371/journal.pone.0245402

**Published:** 2021-01-07

**Authors:** The PLOS ONE Staff

The third author, Yulong Li, is incorrectly noted as the corresponding author. The correct corresponding author is Lei Zhang. Lei Zhang’s email address is: zhanglei@oregonstate.edu. The publisher apologizes for the error.
